# New Potato

**Published:** 1876-05

**Authors:** 


### NEW POTATO.

A GREAT deal has been said for and against the Chinese Yam, or *Dioscorea Batatas*, a species of potato. Some of the agricultural papers have ridiculed it most vehemently. But ridicule is no argument, although it is the common resort of scientific men and fools. We recommend our readers to pay no attention to what anybody says, but experiment for themselves. Dr. WELLINGTON stated to the American Institute of this city, that he had grown a specimen two feet long, and that the largest specimens weighed two pounds and a half, and its yield double that of the common potato. The efforts made to raise it in France had been well rewarded. Mr. FULLER said, the method of culture most successful with him was to cut the vine into pieces, each piece to be buried so as to leave one leaf lying on the surface of the soil. Water it well at the time of planting, and in about four weeks the young potatoes will begin to form. They should be taken up, and kept in dry sand during the winter. One thousand bushels might be raised to an acre, by leaving the plant two or three seasons to fill the ground. The vines will grow fifteen feet long; if for propagation, train them on poles. As it is claimed that a most delicious and healthful food might be produced from them, we advise such of our readers as have the means of trial to make a fair and unprejudiced experiment.

